# Clinico-Pathological Features and Immunohistochemical Comparison of p16, p53, and Ki-67 Expression in Muscle-Invasive and Non-Muscle-Invasive Conventional Urothelial Bladder Carcinoma

**DOI:** 10.3390/clinpract13040073

**Published:** 2023-07-09

**Authors:** Abdulkarim Hasan, Yasien Mohammed, Mostafa Basiony, Mehenaz Hanbazazh, Abdulhadi Samman, Mohamed Fayek Abdelaleem, Mohamed Nasr, Hesham Abozeid, Hassan Ismail Mohamed, Mahmoud Faisal, Eslam Mohamed, Diaa Ashmawy, Mohamed Tharwat, Deaa Fekri Morsi, Abeer Said Farag, Eman Mohamed Ahmed, Noha M. Aly, Hala E. Abdel-Hamied, Doaa E. A. Salama, Essam Mandour

**Affiliations:** 1 Pathology Department, Faculty of Medicine, Al-Azhar University, Cairo 11884, Egypt; 2 Pathology Department, Faculty of Medicine, University of Jeddah, Jeddah 23218, Saudi Arabia; 3 Preventive Medicine, Ministry of Health, Cairo 11516, Egypt; 4 Medixia Global LLC, Sharjah 32223, United Arab Emirates; 5 Histology Department, Faculty of Medicine, Al-Azhar University, Cairo 11884, Egypt; 6 Urology Department, Faculty of Medicine, Al-Azhar University, Cairo 11884, Egypt; 7 Urology Department, King Abdullah Medical City, Makkah 24246, Saudi Arabia; 8 Clinical Oncology Department, Faculty of Medicine, Al-Azhar University, Cairo 11884, Egypt; 9 Pathology Department, Faculty of Medicine, Al-Azhar University, Damietta 34517, Egypt; 10 Pathology Department, Faculty of Medicine, Al-Azhar University, Assiut 71524, Egypt; 11 Pathology Department, Faculty of Medicine, Helwan University, Helwan 11795, Egypt; 12 Pathology Department, Faculty of Medicine for Girls, Al-Azhar University, Cairo 11884, Egypt; 13 Pathology Department, School of Medicine, Badr University in Cairo (BUC), Cairo 11829, Egypt

**Keywords:** urinary bladder neoplasms, TURBT, muscle-invasive bladder cancer, p16(INK4A), TP53 protein, Ki-67 antigen, telehealth

## Abstract

Introduction: The identification of bladder detrusor muscle invasion in urothelial cancer is essential for prognosis and management. We studied the clinical, histological, and immunohistochemical expression of p16, p53, and Ki-67 in urothelial detrusor muscle-invasive bladder cancer (MIBC) and urothelial non-detrusor muscle-invasive bladder cancer (NMIBC) in Egyptian patients. Methods: Sixty-two bladder urothelial cancer cases obtained through TURBT were included and divided into two groups: (MIBC, stage T2) and NMIBC (T1). Tissue blocks were recut and re-examined microscopically; then, the immunostaining of p16, p53, and Ki-67 was performed to compare both groups and evaluate the 13% cut-off for Ki-67, 20% for p53, and p16 intensity in various conditions aided by telepathology technology. Results and conclusion: Hematuria was the main clinical first presentation, with no significant difference between either group. The mean age was 61.6 years, with male predominance (52 males and 10 females). The absence of papillary histological pattern was associated with a higher stage, including detrusor muscle invasion (*p* = 0.000). The overall average percent of p53 immunostaining was 12.9%, revealing no significant difference between MIBC and NMIBC when a cut-off of 20% was implicated. The Ki-67 expression was correlated with higher grade and muscle invasion; however, no association was found with the other two markers’ expression. The negative immunostaining of p16 was associated with low grade and NMIBC in the case of the preservation of the papillary pattern. We recommend further studies on the cut-off of widely used markers and more immunohistochemical and genetic studies on the p16(INK4A), taking into consideration the histological pattern of conventional carcinomas.

## 1. Introduction

Urinary bladder cancer is the 10th most common worldwide cancer type, accounting for 3% of global cancers, with more than 573,000 reported new cases in 2020 [1]. More than 212,000 deaths were estimated worldwide due to bladder carcinoma, which is considered the 13th most frequent cause of cancer death [2]. In Egypt, it is the fourth most common cancer and the fifth highest cause of cancer deaths [3].

Bladder cancer is divided clinically into three disease states: muscle-invasive, non-muscle-invasive, and metastatic cancer; each differs in tumor biology, phenotype, prognosis, and management [1,2].

It can be classified histologically into three types: conventional bladder urothelial carcinoma (UC), accounting for more than 90% of total bladder cancers; divergent differentiation UC (including differentiation into squamous cell carcinoma and adenocarcinoma); and nonurothelial carcinoma (5–20% of bladder carcinomas) [4]. This variation provides important insights into phenotype, biology, treatment, and prognosis.

Transurethral resection of the urinary bladder tumor (TURBT) is the standard surgical procedure in the case of NMIBC. The surgery includes the resection of the entirety of the visible tumors, normal-looking bladder mucosa on the border of the tumor, and muscularis propria (muscle layer) at the base of the tumor. After that, the urologist takes a transurethral resection biopsy of the prostatic urethra (on both sides) and a random biopsy from the apparently normal urothelium in the bladder wall. Finally, if possible, a different operating surgeon inspects the bladder lumen to confirm no tumor remains after the procedure. All resected tissue pieces are sent for histopathological examination [5].

About 75% of UCs are seen as low-grade and NMIBC by the time of diagnosis [6]. Low-grade tumors infrequently progress to invasion (1% and 45%) but frequently recur (31–78%) in five-year follow-up records, therefore having a good prognosis, but high-grade papillary neoplasms may progress to MIBC [7].

Clinicopathological variables, particularly grading and staging systems, have been recognized as important prognostic factors in UCs; however, insights into potential markers predicting the progression and behavior of UC have been developed [8].

There have been many different immunohistochemical markers studied so far in bladder tumors, but none of them are used systematically in clinical practice. Previous studies on UCs were based on heterogeneous samples, with many different methodologies and potential for malignancy that are partially inconsistent [9].

A histological examination is crucial for UC grading and local staging, which will affect the management plan and prognostic value. However, histological assessments have obvious limitations, and hence, ancillary methods for grading, such as mitosis count (or proliferation rate) with the expression of specific immunohistochemical (IHC) markers (mainly MIB-1 “Ki-67”), are studied [10,11].

p53 overexpression is thought to be linked to a worse prognosis of urothelial carcinomas. However, there is much confusion regarding this subject, despite the large number of reports. Previous studies revealed that the late UC stage (invasive type) frequently displays alterations in TP53, PTEN, and ERCC2 genes and pathways [12]. p53 immunohistochemical expression is often used as a surrogate for TP53 mutations. The gene encoding p16 (CDKN2 or INK4a gene) is observed as mutated or downregulated in several malignant epithelial cells, including breast and head, and neck cancers, and also in aggressive subtypes of UCs [13].

A recent study concluded that a positive, especially strong, p16 immunostaining might be diagnostically useful in urothelial tumors where the study found expression of p16 in 100% of the non-invasive papillary UCs. On the other hand, MIBCs showed p16 positivity in only 49% of cases [14]. The use of p16 immunohistochemistry is also considered a surrogate for Human papillomavirus (HPV), and several studies suggested variable cancer association with p16 expression based on ethnicity and geographical variability [15]. In this article, we studied the features of muscle-invasive bladder carcinomas in comparison to non-invasive bladder carcinomas in Egyptian patients at the level of histopathology, and immunohistochemical staining of p16, p53, and MIB-1 (Ki-67) to assess their role in bladder cancer pathology of MIBC versus NMIBC.

## 2. Methods

This retrospective study included sixty-two primary diagnoses of urinary bladder UCs, obtained by means of TURBT in the urology department of our institution, taken by the same urological team for patients diagnosed clinically as a urinary bladder mass depending on specific clinical criteria in the period between January 2019 and February 2020.

Patients were referred clinically with hematuria and bladder masses in 40 patients and irritative symptoms in 22 patients. Solitary small polypoidal masses were en-bloc resected; however, large and/or multiple masses were resected sequentially. A video system for documentation of the findings was obtained, including inspection of the urethra, prostate, and bladder neck, followed by mapping of the bladder pathology.

### 2.1. Histological Examination

Tissue blocks were retrieved from the pathology archives after getting the research ethics committee’s approval. The authors reviewed the clinical notes, histopathology reports, and laboratory archives. Hematoxylin and eosin-stained slides (H&E) were prepared along with multiple unstained slides for immunohistochemistry. All cases were reviewed microscopically by 4 independent histopathologists with external histopathological consultation in Egypt and Saudi Arabia, when indicated, and reclassified as follows: NMIBC group included cases of T1 stage according to the WHO 2016 classification, MIBC group included T2 stage cases. A comparison of those two stage groups was performed.

**Figure 1:**
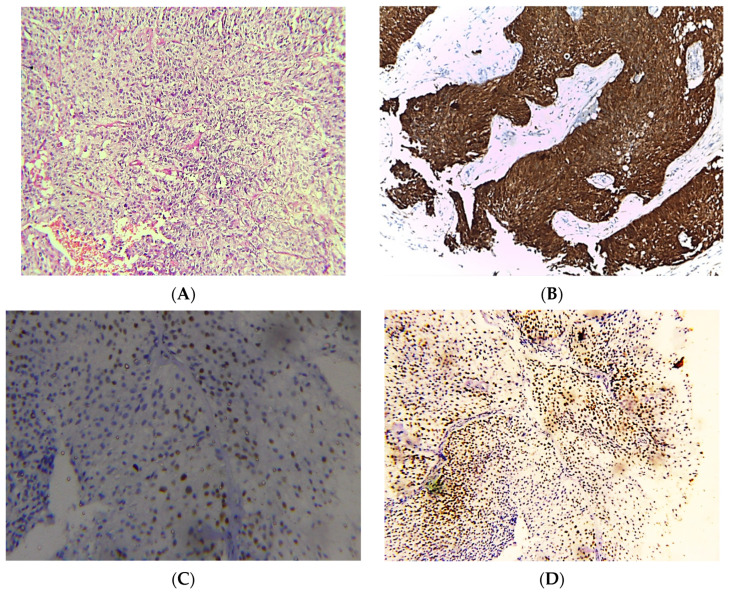
Photomicrographs of NMIBC cases. Histopathological features of urothelial carcinoma reaching laminal propria without muscle invasion (**A**). Immunostaining for p16 in NMIBC showing strong nuclear and cytoplasmic expression (**B**). Immunostaining for p53 in NMIBC showing nuclear expression < 20% of cells (**C**). Immunostaining for Ki-67 in NMIBC showing positive nuclear expression (**D**) (original magnification ×100).

Tumors were reported as papillary urothelial carcinomas according to the histological features of the neoplastic urothelium lining fibrovascular cores, slender papillae with minimal fusion +/− branching on low magnification examination and loss of polarity in addition to mild pleomorphism on medium magnification (Figure 1). However, the latest WHO does not verify this histological classification, but we tried to study this morphological variation in regard to diagnostic utility and immunohistochemical expression. Non-papillary lesions in this study did not show any features of carcinoma in situ within the adjacent mucosa.

High-grade tumors were diagnosed when nuclear pleomorphism, nucleomegaly, irregularity of nuclear contours, readily identifiable mitoses, and clumped chromatin were seen under microscopic examination of at least 5% of the presented non-invasive papillary tumors.

**Figure 2:**
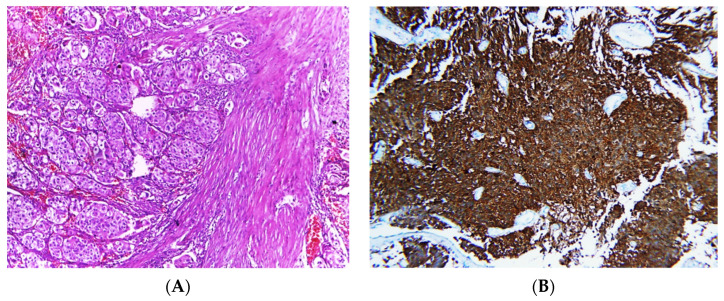

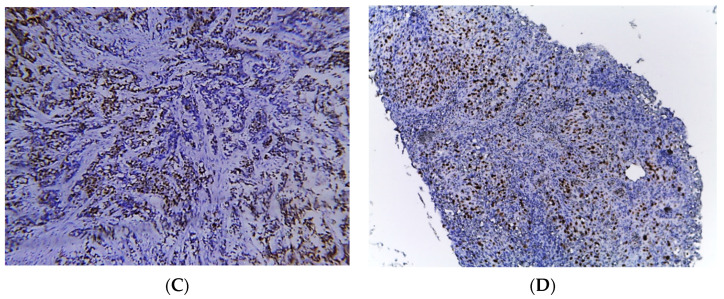
Photomicrographs of MIBC cases. Histopathological features of muscle-invasive urothelial carcinoma (**A**). Immunostaining for p16 in MIBC showing strong nuclear and cytoplasmic expression (**B**). Immunostaining for p53 in MIBC showing positive nuclear expression > 20% of cells (**C**). Immunostaining for Ki-67 in MIBC showing positive nuclear expression (**D**) (original magnification ×100).

Invasive tumors were diagnosed according to specific microscopic criteria; neoplastic cells arranged in irregular nests or single cells invading the lamina propria and muscle layer (muscularis propria) (Figure 2).

### 2.2. Immunohistochemical Study

Prior to IHC, 3–5 µm thick serial tissue sections from representative prepared tissue blocks were taken on positively charged glass slides. IHC was performed at the laboratory of the university hospital using a Ventana BenchMark XT autostainer [Ventana Medical Systems, Tucson, AZ, USA]. Anti-human p16, p53 were performed using a mouse monoclonal antibody [BP53-11] and Ki-67 [clone MM1 mouse monoclonal antibody].

Prepared slides were incubated for 32–40 min at room temperature after the inactivation of the endogenous peroxidases using primary antibodies in addition to the antibodies of negative control. Incubation within polyclonal anti-mouse secondary antibody was conducted for another 30–35 min at room temperature. Primary antibodies + alkaline phosphatase enzyme complexes were incubated for an additional 30 min, and following incubation with 3,3′-diaminobenzidine [Dako Korea LLC] at room temperature for 20 min again, and then the slides were carefully counterstained with Mayer hematoxylin [Merck Millipore, Darmstadt, Germany] for 5–6 min in room temperature. The slides were visualized and examined by the histologists or the pathologists under a light microscope with external consultation when indicated.

#### 2.2.1. p16(INK4A) Scoring

FFPE tissue specimens’ sections were stained for p16(INK4A) expression, and the prepared immunostained slides were evaluated according to Shigehara et al. [16]; depending on these criteria, our results were classified for the statistical analysis into these four groups: negative (−) if less than 5% of tumor cells were negative for nuclear staining, weak (+1) 5–30%, moderate (+2) 30–50%, and strong (+3) >50%, respectively. Positivity means nuclear ± cytoplasmic staining; however, cytoplasmic-only stained cases were considered negative.

#### 2.2.2. p53 Staining

The antibody, p53 protein (DO7, clone NCL-p53-DO7i: Novocastra Laboratories Ltd., Newcastle on Tyne, UK), was a liquid mouse monoclonal used antibody. According to Şen Türk et al. [17], p53 staining (negative < 20% and positive ≥ 20%), we used the average positively stained cells and the 20% cut-off of p53 staining for testing an easier and more accurate tool on the biological behavior of UCs.

#### 2.2.3. Ki-67 Index

The primary antibody used for immunohistochemistry for Ki-67 was as follows (MB67, Neomarkers, US, 1:100, 30 min, tonsil).

Nuclear staining was considered positive for Ki-67 and p53 with the following threshold values: Ki-67 (negative or low < 13% and positive or high > 13%) according to Şen Türk et al. [17].

Evaluation of histology and immunohistochemistry was performed by 4 independent histopathologists using the technology of telepathology to ensure independent and accurate results of the studied tumors. The work was completed with the assistance of Dr. Abdelaleem, who is experienced in telehealth technology and using the platform ihealthcure. A strong emphasis on protecting data privacy in telepathology was placed to ensure the confidentiality and security of patient information, and a comprehensive set of measures has been implemented to safeguard data throughout its lifecycle. This guaranteed the proper functioning of the telecommunication infrastructure, image quality, and data security protocols, ensuring the telepathology technology was optimally utilized and compliant with data privacy regulations.

#### 2.2.4. Exclusion Criteria

Exclusion criteria included benign lesions, recurrent T1 tumors, cases lacking agreement of the histological classification or immunohistochemical evaluation, cases without presented muscle layer in the examined sections, Ta, Tis, T3, T4 stages, non-Egyptian patients, and cases lacking provided main clinical data or tissue blocks.

#### 2.2.5. Ethical Approval

This study was approved by the Research Ethical Review Board of Al-Azhar University, Faculty of Medicine, with ID number: His_395Med.Research00000107. Written informed consent was waived due to the nature of the study, where it is a retrospective study design, and no personal data or pictures are presented.

#### 2.2.6. Statistical Analysis

Statistical analysis of the data provided was performed by the SPSS 21.0 statistical package software (SPSS Inc, Chicago, IL, USA) using the Chi-square test or Fisher exact test. Descriptive analysis was used, including mean ± standard deviations (SD) for quantitative data, frequency tables for the qualitative data, independent sample (*t*-test) for comparing the difference of means in the quantitative data, and Chi-square test for the qualitative data. If the *p*-value was under 0.05, the results were considered statistically significant.

## 3. Results

### 3.1. Clinico-Histological Features

All sixty-two specimens were taken by TURBT using simple cystoscopy. The age ranged from 24 years to 85 (mean ± SD: 61.59 ± 12.580), with 52 males and 10 females. Fifty-nine patients came with hematuria as a main symptom, two patients came with irritative urinary symptoms, and only one patient was discovered accidentally. No significant difference was seen between the main presenting symptom and the tumor grade or muscle invasion.

All included cases were re-evaluated and re-graded according to the WHO classification (2016) with consideration of the histological pattern of papillary configuration, revealing 30 cases of papillary urothelial carcinoma and 32 cases not revealing any papillary features (non-papillary UC) (Figure 1 and Figure 2).

A total of 34/62 (54.8%) cases were described as high-grade, and 28 (45.2%) cases as low-grade UCs. Pathological staging revised according to the new TNM, including 32 (51.6%) of pT1 stage (NMIBC group) and 30 (48.4%) of invasive tumors, pT2 (MIBC group).

Considering pathological stage and histological tumor type, the absence of papillary features correlated with increasing stage and muscle invasion (*p* = 0.000).

### 3.2. Immunohistochemical Expression

No significant relationship was found between immunostaining of p53 (cut-off 20%) and the clinicopathological variables, including age group (>50 years versus ≤50 years old), tumor grade (low versus high), or tumor stage (muscle-invasive versus non-invasive). The average percent of p53 immunostaining was 11.3 for NMIBC and 14.5 for MIBC, with an overall average of 12.9 (Figure 1 and Figure 2).

The Ki-67 index in NMIBC showed an average parentage of 11.8; however, MIBC recorded 23.9 with a significant statistical difference (Table 1). No correlation was detected between the p16 and p53 expressions, or between the p16 and Ki-67 index. Expression of p16 was noted in 54.8% (34 cases) in weak, moderate, and strong positivity, whereas 45.2% (28 cases) was negative for p16 nuclear staining, which reveals no significant difference in the total cases between high versus low grade, invasive versus non- muscle-invasive tumors (Table 2). p16 expression into papillary lesions showed a significant difference between both grades (Table 3) and both stages (Table 4), meaning that the p16 immunostaining density can be used as a diagnostic or potential prognostic marker of papillary urothelial carcinoma.

## 4. Discussion

Urothelial carcinoma of the urinary bladder is one of the most common malignancies in the world, and the prevalence varies across different countries and may differ between the regions of the same country [18,19]. A higher prevalence of urothelial carcinoma of the urinary bladder has been documented in Egypt [3]. Most urothelial carcinomas (around 80%) are superficially invasive tumors but tend to recur, showing a subset progressing to muscle-invasive disease. The remaining cases demonstrate muscle invasion at presentation, which is typically treated with excision, mainly cystectomy and lymph node dissection; as such, MIBC portends a much worse prognosis [20]. The prognosis of patients with UC depends on several factors, including higher stages at the time of diagnosis and the pathologic stage, in which about 2–5% of cases are carcinoma in situ (pTis), 40% are pT1a, 30% are pT1b (no muscle invasion) and 20% are pT2 (muscle invasion) [21]. Therefore, the identification and establishment of specific methods to diagnose and predict the invasion of the tumor is critically important for patient management.

Invasive UCs cystoscopically and grossly can be presented as a sessile, polypoid, fungating, ulcerated and/or infiltrative lesion, however microscopic examination is the cornerstone of detection of invasion and identification of grading [22].

Grading of non-invasive carcinomas is an important factor for prognosis and treatment decisions for the urinary bladder. Two major growth patterns of non-invasive carcinoma exist: papillary and non-papillary (flat). It is supposed that the pathogenesis of these two types of neoplasms occurs through a different molecular pathway. Once the growth pattern of the examined lesion is determined, this lesion is further classified using tiered grading systems that incorporate architectural and cytologic features. The tiered grading system has changed over time [23].

A three-tiered grading system (grades 1, 2, and 3) for papillary urothelial tumors was first introduced in 1973 [24], and revisions occurred in 1998, 1999, 2004, 2016, and 2022 to develop a modern system of grading [25,26,27,28]. Grading of the flat NMIBC follows a similar tiered grading system [1]. In regard to the prognostic role, it is important to note that invasive urothelial carcinoma can be considered “high-grade” by convention, according to some authors, irrespective of the amount of cytological atypia or the depth of invasion [29,30].

Many types of histopathological variants which are associated with MIBC can affect prognosis, and many of those unusual morphologies are commonly associated with biologically aggressive tumors [31]. Urothelial carcinoma with divergent differentiation is the most common variant, including squamous and/or glandular differentiation [32]. Originally, this histologic variant was supposed to have a worse outcome than the conventional urothelial carcinomas, but subsequent stage-for-stage comparisons and analysis showed no significant difference in patients’ survival [32,33]. The micropapillary variant is an aggressive variant and has a distinctive micropapillary feature and resembles ovarian papillary serous carcinoma. Frequently, this variant is seen in MIBC at the time of diagnosis [32].

Another aggressive urothelial carcinoma variant is the nested urothelial carcinoma, characterized by nests of bland-looking urothelial cells without severe atypia, often mimicking the von Brunn nests, making a diagnostic challenge for the histopathologists and carries a poor prognosis due to the late diagnosis with progression and metastases potential in these patients [34]. Plasmacytoid urothelial carcinoma variant is another rare and aggressive urothelial carcinoma, characterized by ovoid cells exhibiting abundant eosinophilic cytoplasm that look like plasma cells that are loosely cohesive in myxoid stroma. Most patients with such aggressive variants present with an advanced stage at the time of diagnosis [35]. Numerous other variants of urinary carcinoma have been described, but the limited number of reported cases in the literature made it difficult to assess the potential prognostic aggressiveness. In this study, no divergent variants were included.

The tumor suppressor p53 protein expression has been shown to have both biological and variable prognostic significance in urothelial carcinomas. Mutations in TP53, the gene encoding the p53 protein, have been well-studied previously in human cancers [36]. Such mutation is a hallmark of the high-grade pathway in the two-pathway model of urinary carcinoma, and the presence of such mutations is associated with tumor progression and decreased patients’ survival in some studies on urinary cancers [37,38]. Some authors used a 10% cut-off value for p53 expression and supposed that using a higher value would have more prognostic relevance [39]. This study aimed to investigate p16 and p53 immunostaining in different stages, grades, and morphology of conventional bladder UCs.

The mean percentage of p53 immunostaining in this study was 12.9, which lies in the average mean reported worldwide in gene mutations, whereas the literature has reported mutations rates in urinary bladder carcinomas ranging from 6% to 61%, and this wide range is likely attributed to variation in tumor grade and muscle invasion [39]. In our study, no statistically significant difference in p53 expression could be seen in regard to MIBC and NMIBC, as well as between both grades (low and high) of both histological types (papillary and non-papillary).

In a recent study, p53 was overexpressed in bladder carcinoma, and its level was increased in high-grade, high-stage, muscle invasion, and metastatic disease [40], consistent with the meta-analysis published by Liao et al. in 2021 and reports about the prognostic value of the p53 in NMIBC [41]. Some authors studied TP53 mutation and concluded that it is more frequent in MIBC compared to NMIBC (35 vs. 70%), and found a correlation with stage, grade, and recurrence of bladder cancer [42,43,44].

Du et al. stated that the TP53 polymorphism influences the risk of bladder cancer initiation; however, the overexpression of the p53 is consistently associated with the increase of T1 NMIBC risk of progression [43]. Given the importance of early diagnosis and treatment, p53 overexpression may be considered a good indication for more aggressive treatment. In spite of these results from many peer studies in different places over the world and during a few decades indicating a prognostic role of p53 immunostaining, our results revealed no significant difference in the p53 expression in MIBC when compared to NMIBC, which comes consistent with few other previous studies including the study conducted by Toll and Epstein reported no statistically significant difference between the invasive and non-invasive bladder tumors concerning IHC staining of p53 [45,46]. Tian and Epstein also studied 26 cases of bladder urothelial carcinomas for p53 and Ki-67 immunohistochemical expression noting that only 2 cases showed a significant increase of p53 staining expression in the invasive compared with the non-invasive tumors [47].

Ki-67 is a well-known nuclear cell proliferation marker that can be visualized and examined through immunohistochemical staining. Ki-67 expression is frequently found in several malignant cancers, including breast, colon, and ovarian cancers. The positive Ki-67 expression, or high index proliferation, has been associated with adverse histological and pathological features with poor recurrence and cancer-free survival, particularly in patients with upper tract urothelial cancer receiving nephroureterectomy [48,49]

Ki-67 investigation in the progression prediction of urothelial carcinomas in both urinary bladder and upper urinary tract lesions gained support from several studies; however, each of them has been faced with a different dilemma [50]. In this study, we divided the expression of Ki-67 into low (<13%) and high and found that there is no significant difference in Ki-67 expression for invasive versus non-muscle invasive lesions in papillary lesions, however in non-papillary lesions proliferation index of Ki-67 showed a significant difference between MIBC and NMIBC. The literature data indicates that the proliferation index has increased in high-grade tumors and in higher stages [51,52]. Studies have established that the Ki-67 index is an independent predictor for progression, recurrence, and response to treatment in MIBC [17,52,53].

In NMIBC, a significant correlation between Ki-67 indexes and tumor recurrence, progression, and cancer-specific survival has been described in several previous studies [43,54,55]. Homozygous deletion at p16 was seen in different stages (T1, NMIBC) and (T2 or more, MIBC) and in different grades of bladder carcinomas, which suggests that the p16 may be involved in the initiation and progression of urinary bladder cancers [56]. Studies show that the p16 genetic status is closely related to the stages of urinary bladder cancer, where the loss of p16 was seen in association with low-grade urothelial carcinoma, while amplified p16 denoted high-grade tumor [57]. In this study, we found a correlation between p16 protein expression and the histological grade of papillary urothelial carcinoma, in addition to a correlation between p16 expression and the muscle invasion of papillary urothelial carcinoma. Previous studies stated that identification of p16 protein expression directly correlates with the tumor behavior and tumor stage where the low-grade tumors and the NMIBCs showed a high expression rate of p16 protein which may help in the process of selecting patients for an early aggressive therapy [13,18]. However, its prognostic significance and the diagnostic utility according to the staining intensity are still unclear, and the studies showed quite different results. Hashmi et al. concluded that high p16 expression was associated with higher grade and muscle invasion [58]. Some authors found that neither p16 gene status nor the p16 protein expression alone can be nominated as an independent predictor for urothelial carcinomas, but combining protein and gene status together provides useful information on such clinical outcomes of these patients [59]. We did not find a significant difference between MIBC and NIBC or between both grades except when differentiating between the papillary group and non-papillary, where the papillary lesions showed a significant difference between p16 expression in high grade compared to the low grade and in MIBC versus NMIBC which may raise the attention of histological pattern significance in conventional UCs. p16 immunohistochemical marker was recently tested in a few types of cancers in Egyptian patients revealing a potential role in carcinogenesis such as colorectal cancers [60], basal cell carcinoma [61], ovarian germ cell tumors [62], and cervical carcinoma [63].

The study limitations that should be taken into consideration in future studies include lack of molecular analyses of the related genes and the inability to study the association between phenotypical IHC and genetic features, the recurrence status for the studied patients was not evaluated and the bladder carcinoma in situ lesions were not assessed.

## 5. Conclusions

A significant difference was seen between p16 protein expression in MIBC and NMIBC and between both tumor grades when the papillary pattern was preserved; however, no association was seen between the status of p16 and p53 expression. The average percentage of p53 immunostaining in this study was 12.9; however, the 20% cut-off value for p53 in conventional bladder carcinomas did not reveal a diagnostic utility for grading or muscle invasion prediction. Ki-67 index showed a significant difference in tumor grade and tumor stage, and a cut-off of 13% may be useful. The first clinical presentation did not reveal a significant difference between MIBC and NMIBC, where we found that more than 95% of all patients came with hematuria.

## Figures and Tables

**Table 1:** Comparison of Ki-67 index (%) in the studied cases.

	Mean	±SD	Range	*p*-Value
Grade				
-Low grade	9.1	9.6	1–30	<0.005
-High grade	17.6	12.8	5–65	
Stage				
-T1 (NMIBC)	8.8	6.4	2–30	<0.005
-T2 (MIBC)	23.9	16.3	3–65	
Pattern				
-Papillary	9.8	6.7	1–30	<0.005
-Non-papillary	17.7	15.3	2–65	

**Table 2:** Comparison of p16, p53, and Ki-67 expression according to grades and stages.

		Total N (%)	p16				*p* Value	p53			Ki-67		*p* Value
			Negative, N (%)	Weak, N (%)	Moderate, N (%)	Strong, N (%)		Negative, N (%)	Positive, N (%)	*p* Value	Low Index, N (%)	High Index, N (%)	
Diagnosis	Papillary UC	30 (48.4%)	13 (21.0%)	1 (1.6%)	7 (11.3%)	9 (14.5%)		26 (41.9%)	4 (6.5%)		23 (37.1%)	7 (11.3%)	
	Non-papillary UC	32 (51.6%)	15 (24.2%)	2 (3.2%)	4 (6.5%)	11 (17.7%)		27 (43.5%)	5 (8.1%)		18 (29.0%)	14 (22.6%)	
	Total	62 (100%)	28 (45.2%)	3 (4.8%)	11 (17.7%)	20 (32.3%)	0.69	53 (85.5%)	9 (14.5%)	0.54	41 (66.1%)	21 (33.9%)	0.07
Grade	High grade	34 (54.8%)	14 (22.6%)	1 (1.6%)	10 (16.1%)	9 (14.5%)		29 (46.8%)	5 (8.1%)		25 (40.3%)	9 (14.5%)	
	Low grade	28 (45.2%)	14 (22.6%)	2 (3.2%)	1 (1.6%)	11 (17.7%)		24 (38.7%)	4 (6.5%)		16 (25.8%)	12 (19.4%)	
	Total	62 (100.0%)	28 (45.2%)	3 (4.8%)	11 (17.7%)	20 (32.3%)	0.24	53 (85.5%)	9 (14.5%)	0.06	41 (66.1%)	21 (33.9%)	0.69
Stage	Stage pT1	32 (51.6%)	15 (24.2%)	0 (0.0%)	8 (12.9%)	9 (14.5%)		27 (43.5%)	5 (8.1%)		24 (38.7%)	8 (12.9%)	
	Stage pT2	30 (48.4%)	13 (21.0%)	3 (4.8%)	3 (4.8%)	11 (17.7%)		26 (41.9%)	4 (6.5%)		17 (27.4%)	13 (21.0%)	
	Total	62 (100.0%)	28 (45.2%)	3 (4.8%)	11 (17.7%)	20 (32.3%)	0.13	53 (85.5%)	9 (14.5%)	0.54	41 (66.1%)	21 (33.9%)	0.1

**Table 3:** p16 expression in papillary urothelial carcinoma according to grade.

			p16				Total
			Negative	Weak Nuclear	Moderate Nuclear	Strong Nuclear	
Grade	High grade	Count	9	0	7	3	19
		% within Grade	47.4%	0.0%	36.8%	15.8%	100.0%
		% within p16	69.2%	0.0%	100.0%	33.3%	63.3%
		% of papillary	30.0%	0.0%	23.3%	10.0%	63.3%
	Low grade	Count	4	1	0	6	11
		% within Grade	36.4%	9.1%	0.0%	54.5%	100.0%
		% within p16	30.8%	100.0%	0.0%	66.7%	36.7%
		% of papillary	13.3%	3.3%	0.0%	20.0%	36.7%
Total		Count	13	1	7	9	30
		% within Grade	43.3%	3.3%	23.3%	30.0%	100.0%
		% within p16	100.0%	100.0%	100.0%	100.0%	100.0%
		% of papillary	43.3%	3.3%	23.3%	30.0%	100.0%

*p* value = 0.02.

**Table 4:** p16 expression in papillary urothelial carcinoma according to stage.

			p16				Total
			Negative	Weak Nuclear	Moderate Nuclear	Strong Nuclear	
Stage	Stage pT1	Count	12	0	7	6	25
		% within Stage	48.0%	0.0%	28.0%	24.0%	100.0%
		% within p16	92.3%	0.0%	100.0%	66.7%	83.3%
		% of Total	40.0%	0.0%	23.3%	20.0%	83.3%
	Stage pT2	Count	1	1	0	3	5
		% within Stage	20.0%	20.0%	0.0%	60.0%	100.0%
		% within p16	7.7%	100.0%	0.0%	33.3%	16.7%
		% of Total	3.3%	3.3%	0.0%	10.0%	16.7%
Total		Count	13	1	7	9	30
		% within Stage	43.3%	3.3%	23.3%	30.0%	100.0%
		% within p16	100.0%	100.0%	100.0%	100.0%	100.0%
		% of Total	43.3%	3.3%	23.3%	30.0%	100.0%

*p* value = 0.03.

## Data Availability

Data are available upon request to the corresponding author.
